# Methyl 4-(1*H*-benzimidazol-2-yl)benzoate trihydrate

**DOI:** 10.1107/S1600536810039188

**Published:** 2010-10-09

**Authors:** Parna Gupta, Soumik Mandal

**Affiliations:** a Department of Chemical Sciences, IISER Kolkata, Mohanpur Campus, 741252 West Bengal, India

## Abstract

The title compound, C_15_H_12_N_2_O_2_·3H_2_O, has been prepared from the reaction of a Schiff base of benzene-1,2-diamine and iron perchlorate at room temperature. The dihedral angle between the benzimidazole ring and the 4-substituted benzene ring is 0.47 (3)°. Hydrogen bonding involving water mol­ecules, imidazole N, imidazole imine H and ester O atoms stabilizes the crystal structure.

## Related literature

For literature on the pharmacological activities of benzimidazole and its derivatives, see: Matsui *et al.* (1994 ▶); Ries *et al.* (2003 ▶). For the 4-nitro analogue, see: Wu (2009 ▶). For the earlier reported structure, see: Bei *et al.* (2000 ▶). For the synthesis of imidazoles and benzimidazoles, see: Du & Wang (2007 ▶).
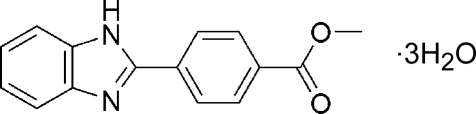


## Experimental

### 

#### Crystal data

C_15_H_12_N_2_O_2_·3H_2_O
*M* *_r_* = 306.32
Triclinic, 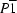

*a* = 6.8308 (3) Å
*b* = 10.8165 (5) Å
*c* = 11.5254 (8) Å
α = 114.718 (3)°
β = 101.718 (4)°
γ = 97.621 (2)°
*V* = 734.41 (7) Å^3^
*Z* = 2
Mo *K*α radiation
μ = 0.11 mm^−1^
*T* = 296 K
0.41 × 0.12 × 0.12 mm

#### Data collection

Bruker APEXII CCD area-detector diffractometer
10457 measured reflections
3194 independent reflections
2873 reflections with *I* > 2σ(*I*)
*R*_int_ = 0.041

#### Refinement

*R*[*F* ^2^ > 2σ(*F* ^2^)] = 0.042
*wR*(*F* ^2^) = 0.142
*S* = 1.02
3194 reflections
218 parameters
H atoms treated by a mixture of independent and constrained refinement
Δρ_max_ = 0.34 e Å^−3^
Δρ_min_ = −0.41 e Å^−3^

### 

Data collection: *APEX2* (Bruker, 2007 ▶); cell refinement: *SAINT* (Bruker, 2007 ▶); data reduction: *SAINT*; program(s) used to solve structure: *SHELXS97* (Sheldrick, 2008 ▶); program(s) used to refine structure: *SHELXL97* (Sheldrick, 2008 ▶); molecular graphics: *SHELXTL* (Sheldrick, 2008 ▶) and *Mercury* (Macrae *et al.*, 2006 ▶); software used to prepare material for publication: *SHELXTL*.

## Supplementary Material

Crystal structure: contains datablocks I, global. DOI: 10.1107/S1600536810039188/ds2060sup1.cif

Structure factors: contains datablocks I. DOI: 10.1107/S1600536810039188/ds2060Isup2.hkl

Additional supplementary materials:  crystallographic information; 3D view; checkCIF report

## Figures and Tables

**Table 1:** Hydrogen-bond geometry (Å, °)

*D*—H⋯*A*	*D*—H	H⋯*A*	*D*⋯*A*	*D*—H⋯*A*
N2—H2⋯O3	0.86	1.99	2.8230 (13)	163
O4—H18⋯O3	0.87 (2)	1.92 (2)	2.7830 (13)	170.1 (17)
O3—H16⋯O2^i^	0.875 (19)	1.910 (19)	2.7840 (12)	177.4 (17)
O3—H17⋯O5^ii^	0.952 (19)	1.745 (19)	2.6932 (13)	173.9 (16)
O4—H19⋯N1^iii^	0.88 (2)	1.89 (2)	2.7614 (14)	171.0 (18)
O5—H20⋯O4^iv^	0.88 (2)	1.95 (2)	2.8291 (14)	174.0 (18)
O5—H21⋯O4^v^	0.88 (2)	1.90 (2)	2.7679 (13)	168.7 (18)
C15—H15⋯O5^vi^	0.93	2.59	3.4009 (15)	146

Symmetry codes: (i) 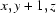; (ii) 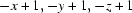; (iii) 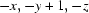; (iv) 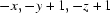; (v) 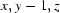; (vi) 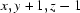.

**Fig. 1.**
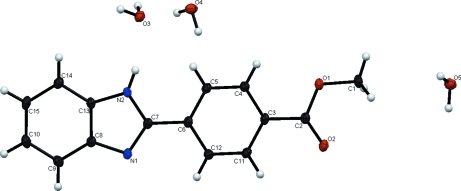
The structure of I with 50% probability displacement ellipsoids and the atom-numbering scheme.

**Fig. 2.**
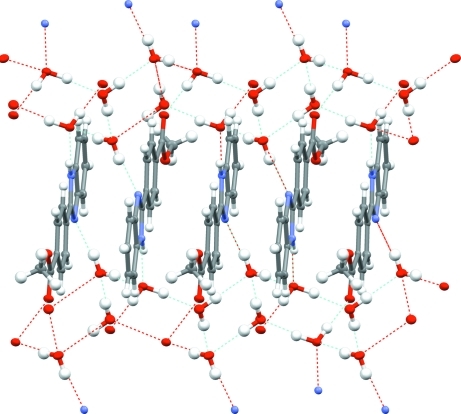
View of compound I along c axis, side-on substructures formed by water molecules.

**Fig. 3.**
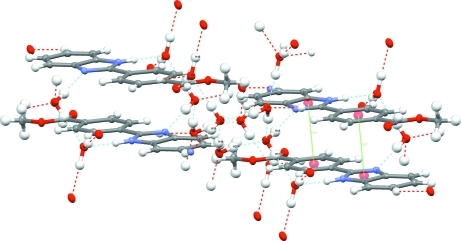
Stacking interaction of compound I.

**Fig. 4.**
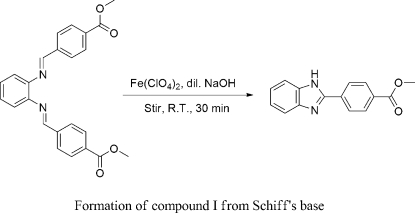
The formation of the title compound.

## supplementary crystallographic information

### Comment

Benzimidazole and its derivatives are important heterocyclic compounds with versatile pharmacological activities and have been reportedly used as antiparasitic, antimicrobial, and antifungal agents (Ries *et al.*, 2003, Matsui *et al.* 1994). They also play very important role in the synthesis of many natural products and synthetic drugs. Herein we report a facile synthesis of compound I from a Schiff's base of 1,2-phenylenediamine using iron perchlorate at room temperature. As a part of our ongoing studies of photophysical properties of metal benzimidazole complexes, the title compound (I) (Fig. 1) was obtained by the reaction of dimethyl 4,4'-(1,2-phenylenebis(azan-1-yl-1-ylidene)bis (methan-1-yl-1-ylidene)dibenzoate and iron perchlorate in presence of dil. NaOH at 298 K (scheme 1). All other previously reported syntheses were done at a higher temperature.

Both the benzimidazole (N1—C7—N2—C13—C8) and methyl benzoate (C3—C4—C5—C6—C12—C11) fragments in this molecule are essentially coplanar similar to the previously reported 4-nitro analogue (De-Hong Wu, 2009). Here, benzimidazole N—H atom does not involve in N—H···N intermolecular hydrogen bonds as in all other cases. This benzimidazole N—H interacts with O3 from one of the water molecules (H17—O3—H17) and one of the hydrogen H16 (from the same water molecule) to form hydrogen bonding interaction with O2 atom of the ester moiety. In addition, N1 atom of benzimidazole ring forms hydrogen bond with the H18 atom of H18—O4—H19 molecule. Another notable hydrogen bonding interaction involves a bridge formation by the oxygen atom O5 in (H20—O5—H21) with two water molecules (H18—O4—H19). The water molecules are contained within channels running parrllel to the *a* axis. However, view along *c* axis shows clearly the side-on substructure formed by water molecules (Fig. 2). Examination of the bond length data reveal that the distances N1—C7 and N2—C7 in the imidazole ring are respectively 1.3321 (15)Å and 1.3638 (14) Å, and is intermediate between the model C—N bond length of 1.48Å and the typical C═N distance of 1.28 Å, indicating partial double-bond character. This can be interpreted in terms of conjugation within the heterocyclic ring system. The bond angles N1—C7—N2 and C5—C6—C12 are 112.56 (10)° and 118.95 (10)° respectively, which is similar to the earlier reported structure (Bei *et al.*, 2000). The imidazole ring (N1—C7—N2—C8—C13) of one molecule and the phenyl ring (C8—C9—C10—C15—C14—C13) of another molecule are approximately parallel with the distance between their centroids being 3.520 (3)Å indicating π-stacking interactions displaying a two-dimensional supramolecular array. The stacking interaction is highlighted in Fig. 3.

### Experimental

A methanolic solution of dimethyl 4,4'-(1,2-phenylenebis(azan-1-yl-1-ylidene)bis(methan -1-yl-1-ylidene)dibenzoate) (synthesized by condensation of 1,2-phenylene diamine and 4-formyl methyl benzoate in 1:2 stoichiometric proportion) 109.40 mg (2.7 mmol) and Fe(ClO_4_)_2_.6H_2_O, 50 mg (1.35 mmol) were mixed and to it was added 5 drops of 5% NaOH solution. The solution immediately changes from yellow to colourless. The solution was further stirred for 30 minutes at room temperature (298 K). The resulting solution was allowed to evaporate slowly at room temperature from which colourless single crystals of the title compound were obtained (Scheme 1).

### Crystal data

**Table:**

C_15_H_12_N_2_O_2_·3H_2_O	*Z* = 2
*M**_r_* = 306.32	*F*(000) = 324
Triclinic, *P*1	*D*_x_ = 1.390 Mg m^−^^3^
Hall symbol: -P 1	Mo *K*α radiation, λ = 0.71073 Å
*a* = 6.8308 (3) Å	Cell parameters from 8743 reflections
*b* = 10.8165 (5) Å	θ = 2.2–40.7°
*c* = 11.5254 (8) Å	µ = 0.11 mm^−^^1^
α = 114.718 (3)°	*T* = 296 K
β = 101.718 (4)°	Rod, colourless
γ = 97.621 (2)°	0.41 × 0.12 × 0.12 mm
*V* = 734.41 (7) Å^3^	

### Data collection

**Table:**

Bruker APEXII CCD area-detector diffractometer	2873 reflections with *I* > 2σ(*I*)
Radiation source: fine-focus sealed tube	*R*_int_ = 0.041
graphite	θ_max_ = 27.0°, θ_min_ = 2.0°
φ and ω scans	*h* = −8→7
10457 measured reflections	*k* = −13→13
3194 independent reflections	*l* = −14→14

### Refinement

**Table:**

Refinement on *F*^2^	Primary atom site location: structure-invariant direct methods
Least-squares matrix: full	Secondary atom site location: difference Fourier map
*R*[*F*^2^ > 2σ(*F*^2^)] = 0.042	Hydrogen site location: inferred from neighbouring sites
*wR*(*F*^2^) = 0.142	H atoms treated by a mixture of independent and constrained refinement
*S* = 1.01	*w* = 1/[σ^2^(*F*_o_^2^) + (0.1051*P*)^2^ + 0.1366*P*] where *P* = (*F*_o_^2^ + 2*F*_c_^2^)/3
3194 reflections	(Δ/σ)_max_ < 0.001
218 parameters	Δρ_max_ = 0.34 e Å^−^^3^
0 restraints	Δρ_min_ = −0.41 e Å^−^^3^

### Special details

**Table:**

Geometry. All e.s.d.'s (except the e.s.d. in the dihedral angle between two l.s. planes) are estimated using the full covariance matrix. The cell e.s.d.'s are taken into account individually in the estimation of e.s.d.'s in distances, angles and torsion angles; correlations between e.s.d.'s in cell parameters are only used when they are defined by crystal symmetry. An approximate (isotropic) treatment of cell e.s.d.'s is used for estimating e.s.d.'s involving l.s. planes.
Refinement. Refinement of *F*^2^ against ALL reflections. The weighted *R*-factor *wR* and goodness of fit *S* are based on *F*^2^, conventional *R*-factors *R* are based on *F*, with *F* set to zero for negative *F*^2^. The threshold expression of *F*^2^ > σ(*F*^2^) is used only for calculating *R*-factors(gt) *etc*. and is not relevant to the choice of reflections for refinement. *R*-factors based on *F*^2^ are statistically about twice as large as those based on *F*, and *R*- factors based on ALL data will be even larger.

### Fractional atomic coordinates and isotropic or equivalent isotropic displacement parameters (Å^2^)

**Table:**

	*x*	*y*	*z*	*U*_iso_*/*U*_eq_	
O1	0.31311 (14)	0.28296 (9)	0.39119 (8)	0.0166 (2)	
O5	0.21253 (15)	0.02407 (10)	0.63276 (9)	0.0209 (2)	
H21	0.155 (3)	−0.050 (2)	0.554 (2)	0.031*	
H20	0.122 (3)	0.076 (2)	0.6355 (19)	0.031*	
O3	0.37646 (14)	0.87010 (9)	0.26610 (9)	0.0176 (2)	
H17	0.523 (3)	0.9011 (18)	0.3003 (17)	0.026*	
H16	0.333 (3)	0.9357 (19)	0.2506 (17)	0.026*	
O2	0.22975 (14)	0.07258 (9)	0.20910 (9)	0.0182 (2)	
O4	0.07060 (15)	0.80876 (9)	0.37434 (9)	0.0207 (2)	
H19	−0.003 (3)	0.723 (2)	0.3151 (19)	0.031*	
H18	0.170 (3)	0.8188 (19)	0.3397 (19)	0.031*	
N1	0.19210 (15)	0.44471 (10)	−0.18336 (10)	0.0133 (2)	
N2	0.28539 (14)	0.64581 (10)	0.00717 (9)	0.0124 (2)	
H2	0.3197	0.7017	0.0912	0.015*	
C1	0.3320 (2)	0.21255 (13)	0.47386 (13)	0.0211 (3)	
H1A	0.2092	0.1397	0.4436	0.032*	
H1B	0.3504	0.2790	0.5647	0.032*	
H1C	0.4488	0.1722	0.4682	0.032*	
C2	0.26140 (17)	0.20014 (12)	0.25945 (12)	0.0135 (3)	
C3	0.24921 (17)	0.28025 (12)	0.18125 (11)	0.0127 (3)	
C4	0.29491 (18)	0.42689 (12)	0.24419 (12)	0.0146 (3)	
H4	0.3289	0.4763	0.3366	0.018*	
C5	0.28948 (18)	0.49866 (12)	0.16823 (12)	0.0146 (3)	
H5	0.3193	0.5962	0.2104	0.017*	
C6	0.23967 (17)	0.42582 (11)	0.02890 (11)	0.0121 (3)	
C7	0.23803 (17)	0.50275 (12)	−0.05091 (12)	0.0120 (2)	
C8	0.21047 (17)	0.55767 (12)	−0.21278 (11)	0.0130 (3)	
C9	0.17985 (19)	0.56017 (13)	−0.33573 (12)	0.0166 (3)	
H9	0.1431	0.4777	−0.4155	0.020*	
C10	0.20643 (19)	0.69051 (13)	−0.33395 (12)	0.0179 (3)	
H10	0.1872	0.6949	−0.4142	0.021*	
C11	0.19577 (18)	0.20677 (12)	0.04230 (12)	0.0150 (3)	
H11	0.1628	0.1092	0.0003	0.018*	
C12	0.19151 (18)	0.27840 (12)	−0.03356 (12)	0.0147 (3)	
H12	0.1567	0.2286	−0.1260	0.018*	
C13	0.26809 (17)	0.68429 (12)	−0.09392 (11)	0.0124 (3)	
C14	0.29457 (18)	0.81564 (12)	−0.09143 (12)	0.0155 (3)	
H14	0.3323	0.8983	−0.0118	0.019*	
C15	0.26175 (18)	0.81641 (13)	−0.21382 (13)	0.0170 (3)	
H15	0.2765	0.9017	−0.2167	0.020*	

### Atomic displacement parameters (Å^2^)

**Table:**

	*U*^11^	*U*^22^	*U*^33^	*U*^12^	*U*^13^	*U*^23^
O1	0.0222 (5)	0.0159 (4)	0.0151 (4)	0.0054 (3)	0.0048 (3)	0.0102 (3)
O5	0.0233 (5)	0.0193 (5)	0.0160 (5)	0.0049 (4)	0.0007 (4)	0.0066 (4)
O3	0.0199 (5)	0.0138 (4)	0.0189 (4)	0.0043 (3)	0.0029 (4)	0.0084 (3)
O2	0.0231 (5)	0.0140 (4)	0.0194 (4)	0.0053 (3)	0.0041 (4)	0.0101 (4)
O4	0.0223 (5)	0.0161 (4)	0.0179 (5)	0.0009 (4)	0.0056 (4)	0.0035 (4)
N1	0.0130 (5)	0.0139 (5)	0.0146 (5)	0.0040 (4)	0.0029 (4)	0.0084 (4)
N2	0.0135 (5)	0.0127 (5)	0.0118 (5)	0.0036 (4)	0.0025 (4)	0.0069 (4)
C1	0.0306 (7)	0.0214 (6)	0.0178 (6)	0.0076 (5)	0.0067 (5)	0.0146 (5)
C2	0.0113 (5)	0.0154 (5)	0.0165 (6)	0.0045 (4)	0.0041 (4)	0.0093 (5)
C3	0.0103 (5)	0.0148 (6)	0.0169 (6)	0.0049 (4)	0.0039 (4)	0.0102 (5)
C4	0.0167 (6)	0.0147 (6)	0.0130 (5)	0.0047 (4)	0.0037 (4)	0.0069 (4)
C5	0.0158 (6)	0.0121 (5)	0.0170 (6)	0.0044 (4)	0.0038 (4)	0.0078 (4)
C6	0.0094 (5)	0.0142 (5)	0.0160 (6)	0.0049 (4)	0.0038 (4)	0.0093 (5)
C7	0.0089 (5)	0.0131 (5)	0.0156 (5)	0.0040 (4)	0.0026 (4)	0.0080 (4)
C8	0.0108 (5)	0.0140 (5)	0.0155 (6)	0.0043 (4)	0.0028 (4)	0.0080 (4)
C9	0.0162 (6)	0.0196 (6)	0.0147 (6)	0.0051 (4)	0.0030 (4)	0.0090 (5)
C10	0.0162 (6)	0.0245 (6)	0.0178 (6)	0.0062 (5)	0.0035 (5)	0.0144 (5)
C11	0.0163 (6)	0.0121 (5)	0.0176 (6)	0.0047 (4)	0.0038 (5)	0.0080 (5)
C12	0.0162 (6)	0.0143 (5)	0.0130 (5)	0.0045 (4)	0.0022 (4)	0.0066 (4)
C13	0.0097 (5)	0.0161 (5)	0.0144 (5)	0.0045 (4)	0.0029 (4)	0.0095 (4)
C14	0.0143 (6)	0.0151 (6)	0.0189 (6)	0.0045 (4)	0.0042 (4)	0.0094 (5)
C15	0.0147 (6)	0.0183 (6)	0.0239 (6)	0.0053 (4)	0.0052 (5)	0.0150 (5)

### Geometric parameters (Å, °)

**Table:**

O1—C2	1.3391 (14)	C4—C5	1.3904 (16)
O1—C1	1.4448 (14)	C4—H4	0.9300
O5—H21	0.88 (2)	C5—C6	1.4015 (16)
O5—H20	0.88 (2)	C5—H5	0.9300
O3—H17	0.952 (19)	C6—C12	1.4049 (16)
O3—H16	0.875 (19)	C6—C7	1.4754 (15)
O2—C2	1.2197 (14)	C8—C9	1.4021 (16)
O4—H19	0.88 (2)	C8—C13	1.4058 (16)
O4—H18	0.87 (2)	C9—C10	1.3882 (16)
N1—C7	1.3321 (15)	C9—H9	0.9300
N1—C8	1.3953 (14)	C10—C15	1.4102 (18)
N2—C7	1.3638 (14)	C10—H10	0.9300
N2—C13	1.3810 (14)	C11—C12	1.3885 (16)
N2—H2	0.8600	C11—H11	0.9300
C1—H1A	0.9600	C12—H12	0.9300
C1—H1B	0.9600	C13—C14	1.3956 (15)
C1—H1C	0.9600	C14—C15	1.3864 (17)
C2—C3	1.4873 (15)	C14—H14	0.9300
C3—C11	1.3966 (16)	C15—H15	0.9300
C3—C4	1.3978 (16)		
			
C2—O1—C1	116.02 (9)	C12—C6—C7	120.53 (10)
H21—O5—H20	101.8 (17)	N1—C7—N2	112.56 (10)
H17—O3—H16	107.0 (16)	N1—C7—C6	125.68 (10)
H19—O4—H18	101.0 (17)	N2—C7—C6	121.76 (10)
C7—N1—C8	104.98 (9)	N1—C8—C9	130.49 (11)
C7—N2—C13	107.37 (9)	N1—C8—C13	109.61 (10)
C7—N2—H2	126.3	C9—C8—C13	119.89 (10)
C13—N2—H2	126.3	C10—C9—C8	117.47 (11)
O1—C1—H1A	109.5	C10—C9—H9	121.3
O1—C1—H1B	109.5	C8—C9—H9	121.3
H1A—C1—H1B	109.5	C9—C10—C15	121.84 (11)
O1—C1—H1C	109.5	C9—C10—H10	119.1
H1A—C1—H1C	109.5	C15—C10—H10	119.1
H1B—C1—H1C	109.5	C12—C11—C3	120.47 (11)
O2—C2—O1	123.50 (11)	C12—C11—H11	119.8
O2—C2—C3	123.61 (11)	C3—C11—H11	119.8
O1—C2—C3	112.89 (10)	C11—C12—C6	120.20 (11)
C11—C3—C4	119.73 (10)	C11—C12—H12	119.9
C11—C3—C2	118.94 (10)	C6—C12—H12	119.9
C4—C3—C2	121.31 (11)	N2—C13—C14	131.59 (11)
C5—C4—C3	119.82 (11)	N2—C13—C8	105.47 (10)
C5—C4—H4	120.1	C14—C13—C8	122.93 (11)
C3—C4—H4	120.1	C15—C14—C13	116.52 (11)
C4—C5—C6	120.82 (11)	C15—C14—H14	121.7
C4—C5—H5	119.6	C13—C14—H14	121.7
C6—C5—H5	119.6	C14—C15—C10	121.34 (11)
C5—C6—C12	118.95 (10)	C14—C15—H15	119.3
C5—C6—C7	120.52 (10)	C10—C15—H15	119.3

### Hydrogen-bond geometry (Å, °)

**Table:**

*D*—H···*A*	*D*—H	H···*A*	*D*···*A*	*D*—H···*A*
N2—H2···O3	0.86	1.99	2.8230 (13)	163
O4—H18···O3	0.87 (2)	1.92 (2)	2.7830 (13)	170.1 (17)
O3—H16···O2^i^	0.875 (19)	1.910 (19)	2.7840 (12)	177.4 (17)
O3—H17···O5^ii^	0.952 (19)	1.745 (19)	2.6932 (13)	173.9 (16)
O4—H19···N1^iii^	0.88 (2)	1.89 (2)	2.7614 (14)	171.0 (18)
O5—H20···O4^iv^	0.88 (2)	1.95 (2)	2.8291 (14)	174.0 (18)
O5—H21···O4^v^	0.88 (2)	1.90 (2)	2.7679 (13)	168.7 (18)
C15—H15···O5^vi^	0.93	2.59	3.4009 (15)	146

Symmetry codes:  (i) *x*, *y*+1, *z*; (ii) −*x*+1, −*y*+1, −*z*+1; (iii) −*x*, −*y*+1, −*z*; (iv) −*x*, −*y*+1, −*z*+1; (v) *x*, *y*−1, *z*; (vi) *x*, *y*+1, *z*−1.
